# A Tutorial on Conducting and Interpreting a Bayesian Independent *T*‐Test Using Open‐Source Software

**DOI:** 10.1111/jan.70122

**Published:** 2025-08-04

**Authors:** Helen Evelyn Malone, Imelda Coyne

**Affiliations:** ^1^ School of Nursing and Midwifery Trinity College Dublin Dublin Ireland

**Keywords:** Bayes factor, Bayesian independent *t*‐test, Bayesian inference, Bayesian parameter estimation, credibility interval, frequentist independent *t*‐test, midwives, nurses, *p* value, tutorial

## Abstract

**Aim:**

To demonstrate a worked‐out example of a Bayesian independent *t*‐test using open‐source software, simulated data, a hypothetical nurse education intervention and a randomised controlled study design. This tutorial explains relevant Bayesian concepts and highlights literature that provides statistically principled justifications for replacing or complementing the frequentist independent *t*‐test with its Bayesian counterpart.

**Design:**

Bayesian *t*‐test analysis tutorial.

**Methods:**

A pedagogical framework was applied.

**Data Source:**

Simulated data generated in Microsoft Excel was uploaded to the Open Science Framework, accessible at: osf.io/4t9gn.

**Results:**

The Bayesian independent *t*‐test in JASP provides: (1) a Bayes factor quantifying the relative evidence for determining which of two competing theories, that is, the null (H_0_) or the alternative (H_1_) hypotheses, better supports the experimental data and (2) the posterior probability distribution, with its median point estimate plus a 95% credible interval, quantifying the magnitude and uncertainty of the effect size estimate.

**Conclusions:**

This article provides a practical method for nursing and midwifery researchers to conduct Bayesian analysis, offering statistical, practical and ethical advantages, including the application of sequential analysis and optimal stopping rules enhancing research efficiency.

**Implications for the Profession and/or Patient Care:**

This article increases awareness of the feasibility and benefits of Bayesian analysis in nursing and midwifery research, emphasising its ease of implementation through open‐source software. Clear step‐by‐step guidance is provided to support its wider adoption and strengthen methodological rigour in nursing and midwifery research.

**Impact:**

Nursing and midwifery research has traditionally relied upon frequentist statistical techniques, based on *p* values and confidence intervals. Bayesian methods can: (a) improve nursing and midwifery decision‐making with probabilistic evidence and (b) reduce publication bias by avoiding binary interpretation of research results.

**Reporting Method:**

The methodology aligns with van Doorn et al. (2021) guidelines for conducting and reporting a Bayesian analysis.

**Patient or Public Contribution:**

No patient or public contribution.

**Summary**

- What does this paper contribute to the wider global clinical community?
  ○ Increases awareness that Bayesian methods are increasingly emerging as a principled approach to data analysis based on probability axioms and the likelihood principle.
  ○ Demonstrates a practical and feasible Bayesian inferential method for analysing two group comparison of means, enabling nursing and midwifery researchers to make probabilistic statistical inferences.
  ○ Provides a hypothetical data set to support teaching and learning, enabling nurses and midwives to practice a Bayesian independent samples *t*‐test.

## 1 Introduction

Historically, nurse researchers have predominantly relied on frequentist statistics, also known as classical statistics, which involve the use of *p* values and confidence intervals for inferential analysis. For example, Anthony's (1996) review of statistical methods in the Journal of Advanced Nursing (JAN) showed that nursing research at that time was based on the frequentist approach. Over two decades later, Malone and Coyne (2017) conducted a similar review in (JAN) and found that nursing and midwifery research continued to rely primarily on frequentist methods, commonly applying a broad range of statistical techniques, including parametric tests, such as *t*‐tests, ANOVA techniques, Pearson's correlation and regression analysis, which assume a normal distribution, and non‐parametric tests, such as the chi‐square test, Wilcoxon signed‐rank, Mann–Whitney, Friedman and Kruskal–Wallis, which do not require distributional assumptions. The reliance on frequentist statistical methods is not unique to nursing and midwifery researchers, as these methods remain the most widely used approach in medical (Ospel et al. 2024) and social sciences research (Kumar 2015), with their dominance evident in major nursing and medical research databases. This reliance is also evident in literature guiding the non‐parametric counterparts to parametric tests in nursing research (Malone and Coyne 2019).

### 1.1 Background: Statistical Approach

The widespread reliance on the *p* value and confidence intervals in frequentist statistics raises the question: why use the Bayesian paradigm? Bayesian methods, such as the Bayes factor (BF) (for comparing the relative evidence for two competing hypotheses) and credible intervals (for estimating effect size) offer principled alternatives based on the axioms of probability (Dienes 2011). Advances in computing make Bayesian methods more accessible, highlighting their statistical and practical advantages over frequentist approaches. Hence, there is a growing recognition across the disciplines of the limitations of frequentist methods, prompting increased interest in Bayesian approaches as a more flexible and informative method.

For decades, proponents of frequentist and Bayesian methods have debated the merits of their statistical approaches. Initially, efforts to improve statistical practices focused on improving the application and interpretation of frequentist methods through clearer guidelines and better education. However, a growing body of literature now highlights the statistical and practical advantages of Bayesian methods over the frequentist approach.

Familiarity with Bayesian statistical techniques should help prepare nurses and midwives, as Bayesian analysis methods are likely to become increasingly prevalent in nursing and midwifery research. Its use is also increasingly emerging in the literature across various disciplines, such as medicine, psychology and social sciences. In nursing, for example, authors Wu et al. (2024) published in BMC Nursing, used Bayesian ANOVA to explore professional identity among a group of nursing students, and Jiang et al. (2024) also published in BMC Nursing, used the Bayesian Independent *t*‐test to examine social anxiety and mobile phone addiction among nursing students.

## 2 Practical and Statistical Benefits of Applying a Bayesian Analysis

Bayesian analysis provides considerable advantages not available with frequentist *p* value null hypothesis statistical testing (NHST). For example, statistical benefits such as: the inclusion of prior knowledge, probabilistic inference, ability to provide evidence for both the null and alternative hypotheses, and practical advantages such as sequential analysis and optimal stopping.

### 2.1 Bayesian Analysis Incorporates Prior Information

The incorporation of prior information is a fundamental component of Bayesian analysis and is represented in the software as the prior probability distribution (Quintana and Williams 2018). It is the distribution of credible effect sizes before experimental data observation and can be informed by previous studies or expert opinion. The prior is updated within the software to the posterior probability distribution by Bayes' theorem after experimental data observation. Prior information places a useful constraint on extreme claims (Wagenmakers, Marsman, et al. 2018), thus stabilising the inference (Gelman et al. 2017).

### 2.2 Bayesian Analysis Does Not Violate the Likelihood Principle

Bayesian inference relies on likelihoods (i.e., the heights or ordinates obtained from the likelihood function) for the observed data and therefore adheres to the likelihood principle. The likelihood function represents the probability of obtaining the observed data given a hypothesis, P(D|H) (Dienes 2011) and indicates how well different hypothesised effect sizes predict the data (Spiegelhalter et al. 2004). The likelihood principle states that all the information in the data relevant to an inference is contained in the likelihood function (Etz 2018, 61). A major strength of the Bayesian approach is its consistency with this principle. In contrast, the frequentist *p* value approach violates the likelihood principle (Berry 1987). This is because the *p* value is a tail‐area integral that includes both the observed data plus more extreme unobserved data representing long‐term hypothetical repeated measurements (Wagenmakers, Love, et al. 2018). The frequentist confidence interval (CI) for effect size is also based on hypothetical, long‐term, unobserved data (Quintana and Williams 2018).

### 2.3 Bayesian Analysis Focuses on the Probability of the Hypothesis Given the Data

Researchers formulate a hypothesis and collect data to test that hypothesis. Bayesian analysis focuses on the probability of the hypothesis given the data—formally stated as P(Hypothesis/Data) or P(H/D). In contrast, *p* value statistics give the inverse probability, which focuses on the probability of the data (and more extreme data) given the null hypothesis and stated as P(Data and more extreme data/null hypothesis). Bayesian inference, therefore, provides a more direct way of testing a hypothesis (Cohen 1994; Dienes 2011; Wagenmakers, Marsman, et al. 2018). This is important because the probability of the hypothesis (theory) is the researcher's primary interest.

### 2.4 Bayesian Analysis Provides Quantifiable Evidence Supporting Evidential Decision‐Making

Decision‐making based on the Bayes factor provides quantification of the relative evidence on a continuous scale for the alternative versus the null hypothesis (Malone and Coyne 2023). Importantly, the Bayes factor can provide three possible evidential states: (1) evidence in favour of the alternative hypothesis, (2) evidence in favour of the null hypothesis or (3) inconclusive evidence. In contrast, the *p* value cannot provide evidence in favour of the null hypothesis (Dienes 2016), that is, it cannot accept the null hypothesis—only reject (Kruschke 2013), or fail to reject it, by applying arbitrarily set cut‐off points, using a binary dichotomous significance criterion (typically *p* < 0.05, 5%; Cohen 1994). Additionally, Bayesian analysis via the posterior probability distribution enables researchers to provide a probabilistic statement of the magnitude and uncertainty of effect size.

### 2.5 Bayesian Analysis Allows Sequential Analysis and Optimal Stopping With Consistent Results

Bayesian sequential analysis enables researchers to monitor data as it accumulates, providing consistent results whether the data is analysed incrementally or as a batch (Wagenmakers, Marsman, et al. 2018). Sequential data analysis allows for optimal stopping. Researchers can continue data collection when evidence is weak or discontinue data collection when evidence is compelling. The Bayes factor strength of evidence decision boundary can be planned based on context and the strength of evidence required. The researcher could, for example, define a stopping rule as follows: that is, data collection will discontinue when the Bayes factor reaches strong evidence, that is, when BF_10_ is > 10 in favour of (H_1_) or when the Bayes factor BF_10_ is < 1/10 favouring (H_o_). In practice, this means that experiments can be more ethically and efficiently managed (Wagenmakers et al. 2016) with better use of resources and the researcher's time. In contrast, the frequentist statistical approach requires pre‐defined sample sizes to control long‐term error rates.

## 3 Learning Objectives

Following this tutorial learners should be able to:

- Define the basic Bayesian concepts relating to a Bayesian independent samples *t*‐test analysis
- Conduct a Bayesian independent samples *t*‐test in user‐friendly, open‐source JASP software
- Report and interpret the results for a Bayesian independent samples *t*‐test

While the Bayesian independent *t*‐test requires a few mouse clicks for JASP to automatically provide the graphical and numeric output results, a more detailed understanding of Bayesian concepts enables researchers to better understand the Bayesian approach and interpret the results more competently.

### 3.1 The Bayesian Independent *T*‐Test

Bayesian counterparts exist for many frequentist tests. The Bayesian independent *t*‐test was selected for this pedagogical example because its frequentist counterpart was identified as the most commonly used parametric test in nursing and midwifery research in a review of statistics published in JAN (Malone and Coyne 2017). Both Bayesian and frequentist independent *t*‐tests are used to compare two independent groups, typically to evaluate the effect of an intervention versus a control using a continuous variable. Both approaches test for differences between two groups using a point null, where the effect size under the null hypothesis (H_0_) is zero. These two statistical approaches differ regarding the alternative hypothesis: for example, the Bayesian approach explicitly defines an alternative hypothesis, which can be a simple hypothesis but is typically a composite of hypotheses (Goodman 1999a) consisting of a range of credible effect sizes (Stefan et al. 2019). In contrast, the frequentist approach does not explicitly define an alternative; instead, it can only reject or fail to reject the null hypothesis.

### 3.2 Output in JASP for the Bayesian Independent *T*‐test

The two main outputs for the Bayesian independent samples *t*‐test conducted in JASP are the Bayes factor (obtained from Bayesian hypothesis testing) and a credible interval, that is, the range of credible effect sizes with its associated median (point estimate) both obtained from the posterior probability distribution.

### 3.3 The Bayes Factor (BF)—Bayesian Hypothesis Testing

**FIGURE 1:**
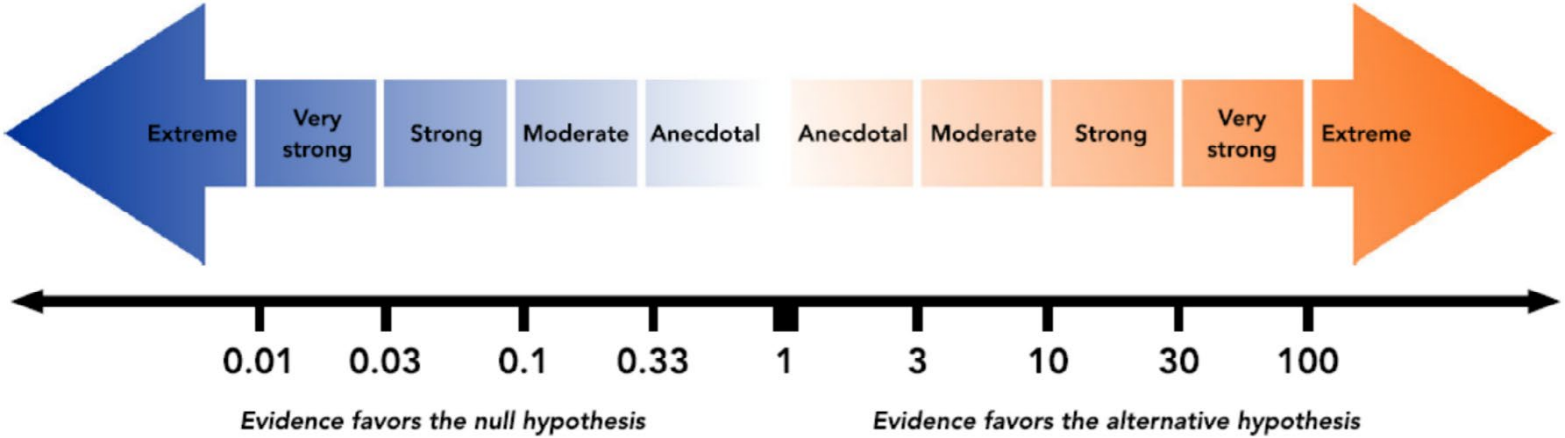
Bayes factor BF_10_ strength of evidence classification scheme adopted in JASP software (with permission from Quintana and Williams 2018).

Bayesian hypothesis testing quantifies the relative evidence of two competing models, that is, (H_0_) and (H_1_) (Wagenmakers, Love, et al. 2018) yielding the Bayes factor defined as the ratio of the marginal (i.e., averaged) likelihoods of the two models (Quintana and Williams 2018), providing evidence as to which hypothesis best predicts the experimental data (Goodman 1999b). For example, a BF_10_ of 20 means that the data is 20 times more likely under (H_1_) than (H_0_) indicating that the observed data is better predicted under (H_1_). The Bayes factor can be interpreted as a graded strength of evidence on a continuous scale for or against the presence of an effect. Figure 1 shows the BF_10_ strength of evidence grading scheme adopted in JASP software (Quintana and Williams 2018). For example, a BF_10_ of 1 indicates equal support for (H_1_) and (H_0_); values between 1 and 3 indicate weak or anecdotal evidence, values between 3 and 10 indicate moderate evidence, and values greater than 10 indicate strong evidence (van Doorn et al. 2021).

The JASP Bayes factor subscripts for the non‐directional Bayesian independent *t*‐test are as follows: BF_10_ indicates Bayes factor evidence in favour of (H_1_) relative to (H_0_), whereas BF_01_ indicates Bayes factor evidence in favour of (H_0_) relative to (H_1_). Note: B_10_ is the inverse of B_01_ (B_10_ = 1/B_01_). The subscripts for the directional Bayesian independent *t*‐test are BF_+0_, and BF_−0_, representing evidence in the positive and negative directions relative to the null hypothesis, respectively.

### 3.4 Effect Size—Bayesian Parameter Estimation

The magnitude and uncertainty of the effect size can be assessed in JASP by examining the posterior distribution of effect sizes under the alternative hypothesis (JASP Team 2024), which can be summarised by a median point estimate and a 95% credible interval (Spiegelhalter et al. 1999).

### 3.5 Bayesian Analysis—Mechanism—Underpinned by Bayes' Theorem

#### 3.5.1 Bayes' Theorem

In the Bayesian independent *t*‐test, Bayes' theorem is applied by the software to obtain the graphical outputs and results. For effect size (parameter estimation) Bayes' theorem (Etz 2018; van Doorn et al. 2021) combines prior beliefs (the prior probability distribution of plausible effect sizes) with the likelihood function, resulting in the posterior probability distribution. This posterior probability distribution is normalised so that the total probability sums to 1.

The ‘parameter estimation’ form of Bayes' theorem can be expressed as (Kruschke 2015, 106):

$$P\left(\delta |\text{Data}\right)\;=\left[P\left(\text{Data}|\delta \right)\times P\left(\delta \right)\right]/P\left(\text{Data}\right)$$

$$\text{Posterior}=\left[\text{Likelihood}\times \text{Prior}\right]/\text{Evidence}$$

where:

- *δ* is the effect size in units of standard deviation of the sampled population (Cohen's *d* units) (van Doorn et al. 2021)
- P(*δ*|Data): is the posterior (the posterior probability distribution of the hypothesised effect sizes *δ* given the experimental data)
- P(Data|*δ*): is the likelihood (evidence for which hypothesised effect sizes are best supported by the experimental data)
- P(*δ*): is the prior—the probability distribution of the hypothesised effect sizes *δ* of the population parameter before seeing the experimental data.
- P(Data): Evidence or marginal likelihood acts as a normalising constant so that the posterior probability distribution sums to a probability of one.

#### 3.5.2 The Prior

(1) The prior, representing plausible effect sizes, is quantified using a prior probability distribution or range of effect sizes that the researcher considers credible before collecting data, and informed by previous studies or expert opinion. For the Bayesian independent *t*‐test, JASP software offers a default prior which is a Cauchy distribution centred on a zero‐effect size (*δ*) with a width‐scale of 0.707 (Goss‐Sampson et al. 2020). This prior has beneficial mathematical properties and has a comparatively small influence on the posterior distribution (van Ravenzwaaij and Etz 2021). The default scale width of the Cauchy distribution in JASP is designed with the Cohen's *d* effect size, in standardised units, making it a scaled distribution that facilitates the generalisation of metrics across different studies. According to Cohen's *d* classification, effect sizes of 0.20 (small), 0.50 (medium) and 0.80 (large) represent effect sizes for comparing independent means (Cohen 1992). In biomedical research greater than or equal to 0.20 (small) but less than 0.50 (medium) effect sizes are typically observed (Kelter 2020). The tails of the Cauchy distribution are heavier than those of a normal distribution allowing for more mass (i.e., probability) for larger effect sizes than allowed by the normal distribution (Rouder et al. 2009). JASP software also provides options to apply a normal or t‐distribution. The JASP software developers recommend not deviating from the default settings unless the consequences are known for customised choices (Wagenmakers, Love, et al. 2018). Deviating from a default prior is acceptable, but it must be justified by explaining why a different prior is more appropriate for the study (van Doorn et al. 2021).

#### 3.5.3 The Likelihood Function

(2) The likelihood function P(Data|*δ*), as a function of the hypothesised effect size *δ* tells the researcher the extent to which different values of the hypothesised effect size *δ* are supported by the observed experimental data. Consequently, a plot of the likelihood function shows the relative plausibility of different values of the hypothesised effect size. It is not a probability distribution; thus, the area under the likelihood function curve is not required to integrate (sum) to one. A plot of the likelihood function is not displayed in the JASP software output window for the independent samples *t*‐test, but it is used with the prior in the background for the calculation of the posterior probability distribution (Spiegelhalter et al. 2004, 18; Peacock and Peacock 2011, 484; Kruschke 2015, 124).

#### 3.5.4 The Posterior

(3) The posterior distribution is a probability distribution that results from combining the prior with the likelihood using Bayes' theorem, enabling the researcher to make probabilistic statements about the effect size (Goss‐Sampson et al. 2020). Since the posterior probability distribution is summarised by a median point estimate and a default 95% credible interval, this enables the researcher to state that there is a 95% probability that the true effect size is within the credibility limits. The posterior probability distribution represents the updated belief about the effect size given all the available evidence and is a synthesis of the evidence of the current study and previous beliefs (Morey et al. 2016).

## 4 Planning Stage

### 4.1 Trial Pre‐Registration

For increased transparency, researchers can pre‐register the study plan on data repositories such as the Open Science Framework (OSF) available at: http://osf.io. Aspects of the Bayesian independent *t*‐test analysis that can be pre‐registered include the specified prior distribution (whether default or customised), Bayes factor decision thresholds, model to be compared (non‐directional or directional), and planned robustness checks for priors. Researchers planning a clinical trial can register it on the prospective registration platform recognised by WHO (World Health Organisation) available at: https://www.who.int/clinical‐trials‐registry‐platform/network. Guiding principles on trial registration and reporting are given by Noyes (2021). Uploading the data to a repository further enhances transparency and enables other researchers to verify the results and test against different prior beliefs.

### 4.2 Guidelines

Guidelines specific to JASP software, for planning, executing, interpreting and reporting a Bayesian analysis are provided by the van Doorn et al. (2021) paper. Discussion points for general Bayesian analysis are provided by (Aczel et al. 2020).

### 4.3 Choosing the Software for the Bayesian Independent *T*‐Test

The relatively recent emergence of user‐friendly, Bayesian‐supported software has improved the accessibility of Bayesian analysis by eliminating the need for users to apply programming skills. For example, in 2017, the long‐established frequentist statistical software IBM‐SPSS (statistical package for the social sciences) introduced Bayesian procedures in version 25. This commercially available software package supports the Bayesian independent *t*‐test, along with other statistical tests, including the Bayesian paired *t*‐test, Bayesian ANOVA, Bayesian correlation and Bayesian linear regression (Peck 2017). Two years earlier, in 2015, with funding from the European Research Council, JASP version 0.7 open‐source software was released, which supports the Bayesian independent *t*‐test, along with other Bayesian techniques, including the Bayesian paired *t*‐test, Bayesian ANOVAs, Bayesian correlation, Bayesian linear regression and contingency tables (Love et al. 2019). Both of these software programmes have since been updated, offering many additional features (IBM‐SPSS 2024; JASP Team 2025). JASP (version 0.18.3) was used for the worked‐out example presented in this article. It offers the benefit of open‐source accessibility and is freely available at: https://jasp‐stats.org/download/.

### 4.4 Research Question: Specifying the Goal of the Analysis

The goal of this study example is to assess the evidence for the effectiveness of a nursing educational intervention programme by applying the Bayesian independent *t*‐test in JASP software. The research question is: Does a new educational programme change the examination score for nursing students? The Bayes factor will be used to assess the evidence for the presence or absence of an effect, and parameter estimation will be used to assess the magnitude and uncertainty of the effect size. More formally, in JASP, the model for the alternative hypothesis is selected in the software, for example, (Group 1 ≠ Group 2), that is, (H_1_: *δ* ≠ 0) (Goss‐Sampson et al. 2020). The null hypothesis model is implicit for the Bayesian independent *t*‐test in JASP, that is, (H_0_: *δ* = 0), where *δ* is the Cohen's *d* standardised effect size (van Doorn et al. 2021).

### 4.5 Data and Sampling Design and Data Entry Into JASP Software

While pedagogical data was simulated for this worked‐out example, a typical nurse education evaluation might recruit a convenience sample of student nurses. In research practice, participants in a randomised controlled trial (RCT) are randomly allocated to either an intervention or control group (Bench et al. 2013). For the pedagogical data, a random normally distributed data set for the two groups, with equal variance, was simulated in Microsoft Excel using the function = NORMINV(RAND(),73,10) for the intervention group (*n* = 100) and = NORMINV(RAND(),68,10) for the control group (*n* = 100). Total (*N* = 200). The simulated order of the controls and treatment groups was also randomised to obtain a typical Bayes factor sequential plot. The generated data was imported as a batch into JASP as an Excel.csv file, but data can also be manually entered into the software in long format with one column for group labels and another column for the scores. The simulated data for this illustrated example was uploaded to the Open Science Framework and is publicly accessible at: osf.io/4t9gn.

### 4.6 Choosing a Directional Instead of a Non‐Directional Bayesian Independent *T*‐Test

The Bayesian independent samples *t*‐test in JASP software facilitates a directional or non‐directional test. A theoretically justifiable reason should be given for the choice between a directional or non‐directional *t*‐test (van Doorn et al. 2021). The intervention for the illustrated example may increase or decrease examination scores. As such, for the Bayesian independent samples *t*‐test presented in this worked‐out example, a non‐directional (two‐sided) test was conducted (i.e., Group 1 ≠ Group 2), which does not assume a specified direction for a difference in examination scores. However, during the planning stage, a directional Bayesian independent samples *t*‐test (i.e., Group 1 > Group 2) could be selected in the software if the researcher's hypothesis specified that the intervention can only increase examination scores.

### 4.7 Sample Size Considerations and the Stopping Rule

Unlike the frequentist approach, the determination of a fixed sample size is not a strict pre‐requisite for Bayesian analysis (van Doorn et al. 2021). However, a sufficient sample size remains important to ensure meaningful results and conclusions. The Bayesian approach offers flexibility in sample size by allowing for continuous monitoring of the Bayes factor evidence as it accumulates and the application of an optimal stopping plan. In research practice, the sampling plan can specify that data collection will stop once the Bayes factor reaches a pre‐decided decision boundary. For example, data collection can be chosen to stop if the Bayes factor crosses the pre‐determined decision boundary BF_10_ > 10 supporting the alternative hypothesis (H_1_) or if the Bayes factor crosses the decision boundary BF_10_ < 1/10 supporting the null hypothesis (H_0_). If the BF_10_ does not cross either decision boundary (i.e., 1/10 < BF_10_ < 10), the evidence can be deemed inconclusive and a decision withheld, suggesting the need for a larger sample size (Schönbrodt et al. 2017). The sampling plan can combine two specifications, that is, data collection will stop when either a maximum sample size (N) is reached or the pre‐determined Bayes factor boundary is reached, whichever comes first (van Doorn et al. 2021). Using more stringent Bayes factor decision thresholds reduces the chance of making early decisions based on weak evidence, which can lead to false positives or false negatives. Bayes factor boundaries do not need to be symmetrical; for example, the upper boundary could be a BF_10_ of 10 and the lower boundary could be a BF_10_ of 1/3, depending on the researcher's concern with false positives or false negatives levels. For this pedagogical illustration, the final BF and credible interval results were assessed for the simulated data set *N* = 200. With a sufficiently large sample size, the Bayes factor is guaranteed to converge to the correct boundary, providing compelling evidence in favour of either the null or alternative hypothesis (Schönbrodt et al. 2017). Using a larger sample size helps achieve lower rates of misleading evidence and greater precision of the parameter estimate of an effect size given by the credible interval (Stefan et al. 2019). Details on how to plan for compelling evidence are provided by (Schönbrodt and Wagenmakers 2018). Compared with an optimal anticipated effect size estimate in NHST, the sequential Bayes factor design typically requires 50% to 70% smaller sample sizes to conclude that an effect size exists while maintaining the same or lower long‐term rate of incorrect inferences (Schönbrodt et al. 2017). The most common applied design plans use directional hypotheses, that is, one‐sided tests (Schönbrodt and Wagenmakers 2018). For ethics and/or funding application requirements, researchers conducting a directional Bayesian independent *t*‐test can use an open access, user‐friendly Shiny app available at: http://shinyapps.org/apps/BFDA/ to inform Bayesian sample size calculations and assess the probabilities of misleading evidence (i.e., false positives and false negatives). Sample sizes for both fixed N and sequential designs can be assessed in the shiny app. It also enables researchers to set a probability to determine the expected sample size to reach a pre‐determined decision boundary. The shiny app for the Bayesian independent *t*‐tests requires the researcher to input an anticipated effect size estimate.

## 5 Implementing the Plan

Data are entered into the software as described earlier.

### 5.1 Descriptive Statistics

In order to display and examine the characteristics of the samples—click ‘'Descriptives’ on the JASP software menu, drag examination scores to the variable pane, drag group to the split pane, click ‘statistics’ and select the descriptive statistics of interest which display in the output pane.

### 5.2 Data Checking for Normality and Equal Variance—Visual and Formal Checks

The assumptions for the parametric Bayesian independent *t*‐test are the same as for its frequentist counterpart: the dependent variable, measured on a continuous scale, is approximately normally distributed with equal variances, absence of outliers and participants are randomly assigned to independent groups. JASP software offers multiple methods to check normality and homogeneity of variance, allowing researchers to choose the most suitable approach (JASP Team 2024). These methods include visual and/or formal statistical approaches. Visual checks for data approximation of normality are available within the software under Descriptives/Basic plots and selection of either a ‘Q‐Q plot’ or selecting ‘Distribution plots’ to display a Histogram with density and rug marks. Alternatively, a hybrid raincloud plot (available via the *T*‐Test tab/Bayesian Independent Samples *T*‐Test) can be selected to visually check the assumptions of approximate normality, equal variance and outliers. To implement formal statistical checks assessing normality and equal variance within the software: click *T*‐Test, Classical Independent Samples *T*‐Test, drag Examination Score to Dependent Variables pane and Group to Grouping Variable pane. Then under ‘assumption checks’ select normality for the Shapiro–Wilk test and under ‘Equal variance’ select Levene's test for equality of variance. Results display automatically.

#### 5.2.1 Options in JASP When the Bayesian Independent t‐Test Assumptions Are Not Met

When assumptions of normality and/or equal variance are violated, the researcher can apply a transformation of data (common transformations include log values or square root values) (Goss‐Sampson et al. 2020); these data transformations are available within ‘data edit’ in JASP. The JASP Bayesian Mann–Whitney, can also be considered because it is a non‐parametric test which is robust to non‐normality (van Doorn et al. 2021). Alternatively, if the groups cannot be assumed to have equal variance with absence of outliers the ‘Bayesian robust *t*‐test’ can be considered (van Doorn et al. 2021) and is available under the ‘Robust T‐test’ tab within the JASP software. It is a useful test because it uses model‐averaging, which can manage data that has either equal or unequal variances and is robust to outliers (Maier et al. 2024). The Bayesian robust *t*‐test, compared to Bayesian independent *t*‐test has the limitation that it is not as straight‐forward to apply and requires a more advanced level of Bayesian knowledge.

## 6 Steps for Conducting the Bayesian Independent *T*‐Test in JASP

Assuming that the dependent variables in each group are approximately normal distributed with equal variance, the test is conducted as follows:

- Click the *T*‐Test menu and select Bayesian Independent Samples *T*‐Test
- Drag ‘Examination score’ into the ‘Dependent Variables’ pane, drag ‘Group’ into the ‘Group variable’ pane
- Under Alternative Hypothesis: select group 1 ≠ group 2
- Under Bayes factor: select BF_10_
- Under Tests: select Student
- Click ‘Prior and posterior’ and ‘additional information’ then JASP can also report Bayesian parameter estimation results. Retain the default 95% credible interval
- Select Bayes factor Robustness check
- Select Sequential analysis
- Retain the default prior, that is, Cauchy scale width 0.707

The blue icon with the letter ‘i’ on the top of the left pane in JASP provides information for this analysis.

The two main outputs that display in the JASP output pane for the Bayesian independent *t*‐test include:

- The Bayes factor and the posterior probability distribution summarised by a median point estimate and a 95% credible interval for the effect size.

## 7 Results With Supporting Explanations

### 7.1 Data: Descriptive Statistics and Checks for Normality and Equality of Variance and Outliers

**FIGURE 2:**
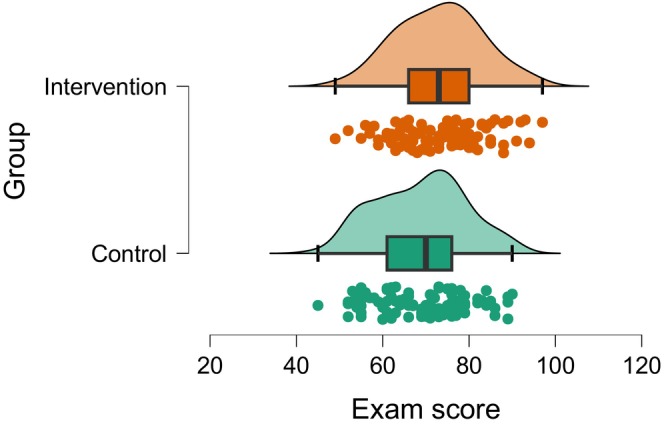
Raincloud or hybrid plots displaying density curves, boxplots and jitter dot plots.

Table 1 presents the intervention study's descriptive statistics for the observed data. Figure 2 shows the raincloud plot, a visual representation of the data, with density curves, boxplots and jittered dot plots, which indicate approximate normality, approximate equal variance and an absence of outliers (i.e., no dots beyond the boxplot whiskers). The formal statistical results, that is, the Shapiro–Wilk test statistic for normality yielded a (*p* value = 0.976, NS) and (*p* value = 0.088, NS; for the intervention and control group), respectively. The Levene test statistic F for equality of variance between the two groups, yielded a (*p* value = 0.499 NS). Both of these tests yielded non‐significant (NS) results (*p* > 5%) indicating consistency with the assumptions of normality and equal variance (JASP Team 2024).

**TABLE 1:** Descriptive statistics for the intervention and control group.

	Group	*N*	Mean	SD	SE	Min.	Max.
Examination score	Intervention	100	73.170	9.937	0.994	49.0	97
	Control	100	68.820	10.318	1.032	45.0	90

*Note:* Standard deviation (SD) pooled: Square root [(9.937^2^ + 10.318^2^) /2] = 10.13.

### 7.2 The Bayesian Independent *T*‐Test Results—The JASP Output Pane

#### 7.2.1 Bayes Factor Results for the Two Competing Models

**FIGURE 3:**
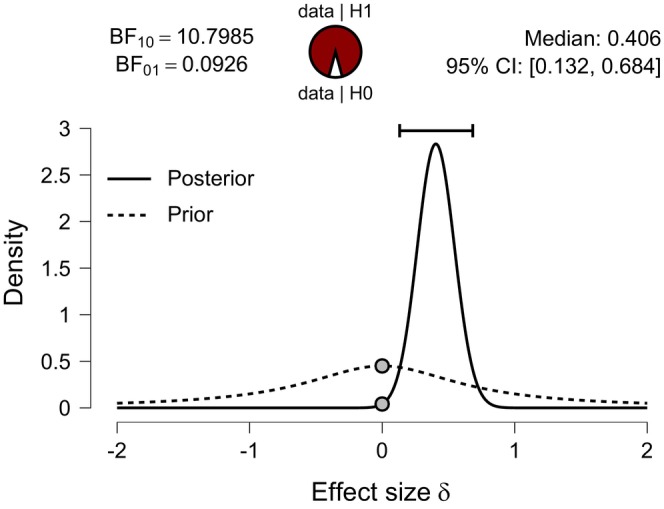
JASP non‐directional Bayesian independent samples *t*‐test results.

Figure 3 displays the JASP output pane providing the results for the Bayesian independent *t*‐test (non‐directional). The analysis yielded a BF_10_ of 10.8. This BF_10_ can be interpreted as indicating that the alternative hypothesis (H_1_) is 10.8 times better at predicting the observed experimental data than the null hypothesis (H_0_), supporting the theory that the educational intervention changed examination scores. The Bayes factor strength of evidence BF_10_ is shown visually (shaded H_1_: unshaded Ho) in odds form (i.e., 10.8:1) in the JASP pizza plot. Figure 3 also displays two grey dots, indicating the density values of the prior and posterior distributions at zero‐effect size (Goss‐Sampson et al. 2020). The ratio of the densities (i.e., heights) at these dots equals the Bayes factor and is known as the Savage‐Dickey density ratio (Wagenmakers, Love, et al. 2018). Figure 1 Bayes factor BF_10_ strength of evidence classification scheme adopted in JASP categorises the BF_10_ of 10.8 as strong evidence in favour of (H_1_) (Quintana and Williams 2018). The strength of evidence required by the researcher should be context‐based (Aczel et al. 2020).

#### 7.2.2 Bayes Factor Sequential Plot

**FIGURE 4:**
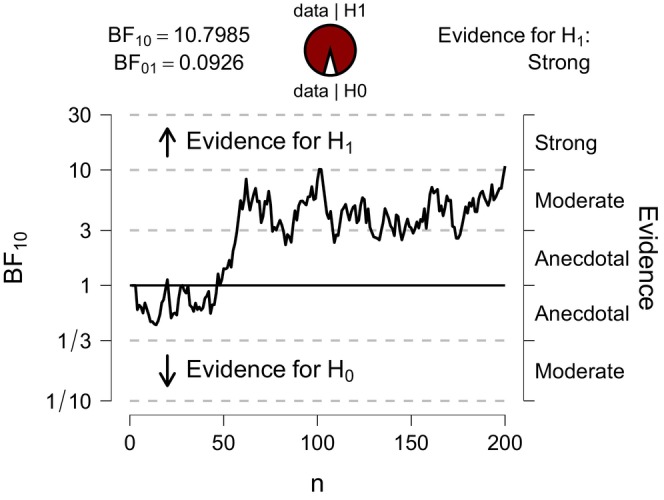
Bayes factor sequential analysis plot.

Figure 4 displays the sequential analysis plot generated by JASP, illustrating how the Bayes factor varied with increasing sample size. Sequential plots enable researchers to monitor the Bayes factor's support for either the alternative hypothesis or the null hypothesis relative to pre‐determined decision boundaries. This facilitates the application of the stopping rule, which allows discontinuation of data collection once the Bayes factor crosses the predefined decision boundary. In JASP software, for the Bayesian independent *t*‐test, the Bayes factor on the sequential plot begins at 1, reflecting equal evidence for the two competing hypotheses. The end‐point Bayes' factor for a sample size N does not depend on the order of data entry, that is, if the order of the data is changed, the sequential plot will show a different path but will arrive at the same end point, that is, giving the same Bayes' factor result. The conclusion is drawn on the totality of the evidence at the stopping point. Note: the increasing trend of the BF_10_ in the sequential plot, including its peaks and troughs, would be more pronounced on a non‐log BF scale. For the intervention study, the BF was assessed for a sample size *N* = 200. Figure 1 shows that the end‐point Bayes' factor of 10.8 represents strong evidence for H_1_. If the sequential plot failed to reach the pre‐determined decision boundary, the Bayes factor evidence can be reported as inconclusive. If feasible, and ethically approved, the researcher may recruit additional participants and choose to continue data collection and monitoring of the Bayes factor.

#### 7.2.3 Robustness of the Bayes Factor to Different Cauchy Prior Widths—BF Sensitivity Check

**FIGURE 5:**
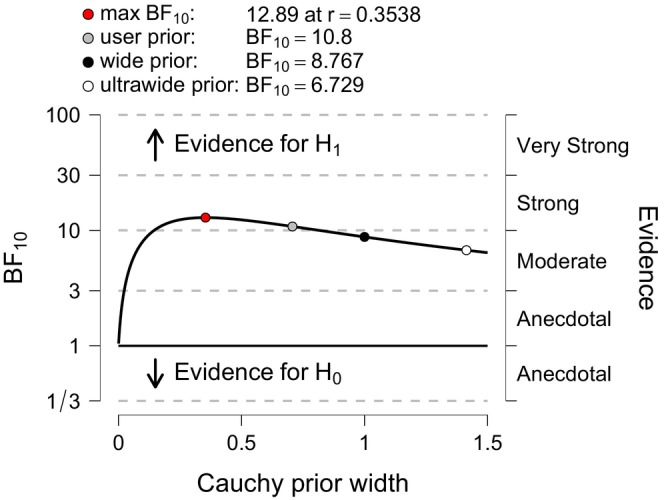
Bayes factor robustness plot.

The Bayes factor can be sensitive to the width of the prior distribution. An assessment of the sensitivity of the Bayes factor to changes in the Cauchy prior width was assessed by selecting the Bayes factor robustness option in the software. The robustness analysis determines whether the Bayes factor (BF_10_ = 10.8) remained consistent across a range of plausible Cauchy prior widths, assigned by the software (Wagenmakers, Love, et al. 2018). Figure 5 displays the result for BF robustness analysis for the intervention study indicating that the BF_10_ was reasonably robust, that is, showed no dramatic changes and stayed within the range of moderate to strong evidence when the Cauchy prior width in Cohen's *d* units is varied from small (*r* = 0.2) to ultrawide (*r* = 1.41) widths.

#### 7.2.4 Robustness of the Bayes Factor Results to Normality

As a non‐parametric test, the Mann–Whitney does not require the data to be normally distributed.

To check for robustness to normality, a Mann–Whitney test was also conducted in JASP using the 1000 samples default and a seed set to 1. The Mann–Whitney test result yielded a BF_10_ of 7.67 indicating moderate evidence (based on the JASP, BF classification scheme, see Figure 1) with a comparable median (0.401) and credible interval [0.12, 0.68] to the Bayesian independent *t*‐test results.

## 8 Supplementing the Bayes Factor Result With Posterior Model Probabilities

Posterior model probabilities can serve as a valuable and intuitive metric for medical diagnosis and health policy decision‐making. Researchers can optionally supplement the BF result by manually calculating posterior model probabilities.

Table 2 shows how to calculate the posterior model probabilities in two simple steps:

**TABLE 2:** Calculation of posterior model probabilities from the Bayes factor with neutral prior odds 1:1.

BF_10_ = 10.8		
Prior belief	Manual calculations	Updated belief
Prior model odds H_1_: Ho 1:1	Posterior model odds	Posterior model odds H_1_: Ho 10.8: 1
	Step 1 Posterior model odds are given by: Posterior model odds = Bayes factor × prior model odds^a^ => 10.8 × 1 = 10.8 … Equation (1) Posterior model odds can be visualised in the pizza plot as the ratio of shaded to unshaded areas (10.8:1)	
Prior model Probabilities H_1_ versus Ho 50% versus 50% (more formally) P(H_1_) versus P(Ho)	Prior and posterior model probabilities	Posterior model Probabilities H_1_ versus Ho 91.5% versus 8.5% (more formally) P(H_1_/D) versus P(Ho/D)
	Step 2 Probabilities = odds / (odds +1)… Equation (2) Therefore: Prior model probabilities for H_1_ and Ho are given by: prior model odds/(prior model odds +1) => 1/ (1 + 1) = 0.50 (50%) Posterior model probability for H_1_ is given by: posterior model odds/(posterior model odds +1) => 10.8/(10.8 + 1) = 0.915 (91.5%) Posterior model probability for Ho is given by the complement of 91.5% => 8.5% The shaded portion of the pizza plot (91.5%) provides a visual representation of the posterior model probabilities for H_1_ while the remaining unshaded portion (8.5%) reflects the probability for Ho	

*Note:* Conclusion: Prior probability for H_1_ increased from 50% to a posterior probability of 91.5%. Prior probability for Ho decreased from 50% to a posterior probability of 8.5%. More sceptical or optimistic prior odds than 1:1 can be incorporated into Equation 1, if required.

^a^ Neutral prior model odds of 1:1 are assigned in JASP software for the Bayesian independent test.

Step 1: The Bayes factor is inserted into the odds form of Bayes' rule (Equation 1) (Goodman 1999a; Dienes 2011; Goss‐Sampson et al. 2020).

(1) Posterior model odds=BF×prior model odds…

Step 2: The posterior model odds can then be converted to posterior model probabilities using (Equation 2) (Goodman 1999a; Gerstman 2013).

(2) $$\text{Probabilities}=\text{odds}/\left(\text{odds}+1\right)\ldots$$

The posterior probability for H_0_ is the complement of that for H_1_.

For the Bayesian independent samples *t*‐test in JASP, neutral prior odds of 1:1 on each model are assigned by default. With neutral prior model odds, the Bayes factor is numerically equal to the posterior model odds (van Ravenzwaaij and Etz 2021). Researchers can apply more sceptical or enthusiastic prior beliefs into (Equation 1), if required (Goodman 2005; Kass and Raftery 1995; Faulkenberry et al. 2020).

### 8.1 Posterior Probabilities Results

Table 2 outlines how the posterior model probabilities were manually calculated for the study to assess probabilistically the change in belief between the competing models H_1_ and H_0_ using neutral prior model odds of 1:1. Applying (Equation 1) yielded posterior model odds of 10.8:1, which converted via (Equation 2) to posterior model probabilities of 91.5% for H_1_ and 8.5% for H_0_. As a result, belief in H_1_ increased from 50% to 91.5% and belief in H_0_ decreased from 50% to 8.5% after observing the data. The posterior probabilities provided probabilistic support for the theory that the intervention resulted in a change in examination scores favouring the belief in H_1_.

### 8.2 Sequential Posterior Model Probabilities Plot

**FIGURE 6:**
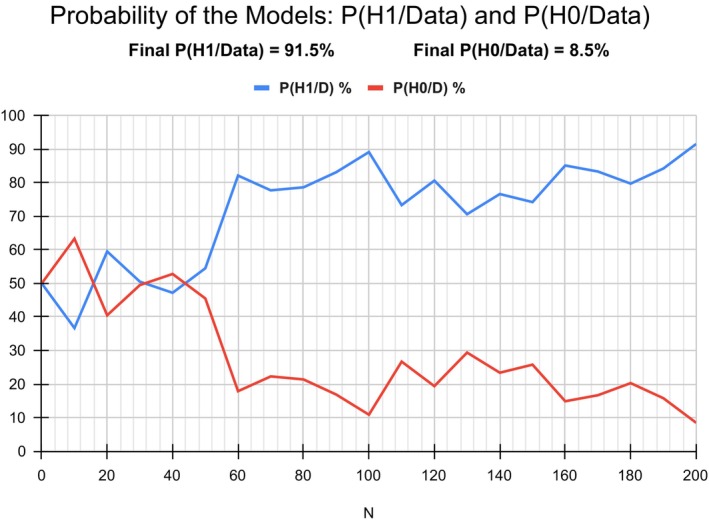
Posterior model probabilities plot (%) displayed sequentially for every 10 data points.

Although currently not available directly within the JASP software, a sequential model probability plot was created in Excel to visualise how the plausibility of each model changes as the sample size increases. Figure 6 shows the posterior model probabilities plot (with prior model odds of 1:1) calculated for each Bayes factor value corresponding to every 10‐participant interval up to *N* = 200. The resulting plot illustrates the increasing belief in H_1_ and a decline in belief in H_0_ as the sample size increases.

## 9 Bayesian Parameter Estimation: Standardised Effect Size and Credible Intervals

Figure 3 shows the effect size estimate obtained for the non‐directional Bayesian independent *t*‐test for the educational programme intervention. The median standardised effect size was *d* = 0.406 (in Cohen's *d* units), with a 95% credible interval, that is, uncertainty range from [0.132 to 0.684]. In JASP software, the 95% credible interval is computed as the central 95% of the posterior distribution for the effect size (Faulkenberry et al. 2020) using an equal‐tail interval ETI. This means that 2.5% of the posterior mass lies below the lower bound and 2.5% above the upper bound (limit). Thus, the median and credible interval limits for the standardised effect size (Cohen's *d*) can be converted to absolute units by multiplying them by the pooled standard deviation of the two groups (see Table 1; Kruschke 2015, 343). Thus, in absolute units, the median (50% percentile) is 4.1, and the uncertainty as expressed by the 95% credible interval ranges from [1.3 to 6.9], that is, [2.5% and 97.5% percentiles], respectively (Kruschke 2015, 342).

## 10 Understanding Directionality in the *T*‐Test Results

**FIGURE 7:**
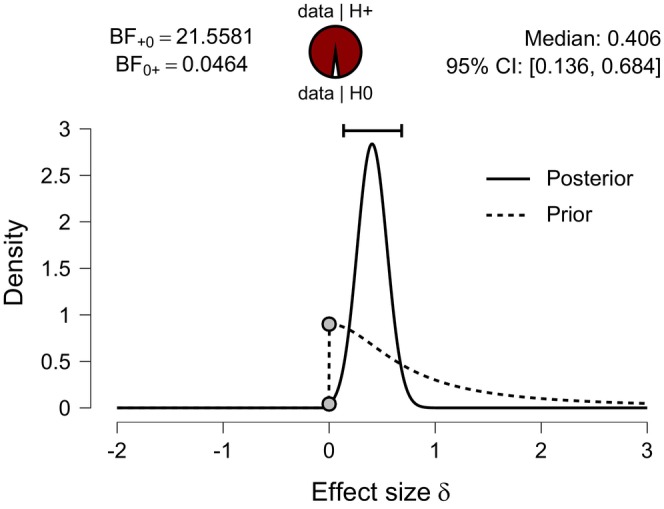
Directional Bayesian Independent samples *t*‐test in JASP.

Researchers who believe that the intervention can only increase examination scores could, in the first instance, justifiably have selected the directional test (i.e., Group 1 > Group 2) option in the software (more formally H_1_: *δ* > 0). For pedagogical reasons, a directional Bayesian independent *t*‐test was also conducted for the intervention study data. Figure 7 shows the JASP output for the directional Bayesian independent test and shows that the prior distribution is restricted to the pre‐specified direction. The credible intervals and median effect size for the directional test (0.136–0.684, *δ* =0.406) when compared to the non‐directional test (0.132–0.684, *δ* = 0.406) are practically equivalent. However, the non‐directional test yielded a BF_10_ = 10.79, which approximately doubled to BF_+0_ = 21.55 when the directional test option is chosen. This increase is reasonable because, while the prior mass was symmetrical about zero for the undirected test, it is now totally concentrated in the positive directions and thus more in agreement with the observed experimental data (Morey and Wagenmakers 2014; Jamil et al. 2017).

## 11 The Summary Report—Example

*Introduction*: This report presents the findings of a Bayesian independent *t*‐test conducted using simulated data, a hypothetical nurse education intervention and an RCT design, to determine whether there was evidential support for an examination score change for a new nurse educational intervention and the probable size of the effect. The analysis was performed using the open‐source JASP software (Version 0.18.3) using the software's objective default Cauchy prior with a scale width of 0.707.

*Descriptive Statistics*: Table 1 summarises the descriptive statistics for the observed examination scores. Intervention Group: Mean = 73.2, Standard Deviation = 9.9, Standard Error = 0.99, Minimum = 49.0, Maximum = 97.0. Control Group: Mean = 68.8, Standard Deviation = 10.3, Standard Error = 1.0, Minimum = 45.0, Maximum = 90.0.

*Data Visualisation and Assumption Testing*: Figure 2 presents a visual representation of the data as a raincloud plot. This visualisation suggests approximate normality, approximate equal variance and no apparent outliers (i.e., no data points beyond the boxplot whiskers). Statistical assumption tests further confirmed these observations: Both the Shapiro–Wilk test for normality and the Levene's test for equality of variance yielded non‐significant *p* values (*p* > 5%) which are consistent with the assumption of normality and equal variance (JASP Team 2024).

*Evidence for the effect* (*Bayes Factor*): Figure 3 presents a non‐directional Bayesian independent samples *t*‐test with a BF_10_ of 10.8, providing evidence that the experimental data is 10.8 times more likely under the alternative hypothesis (H_1_) than under the null hypothesis (H_0_). In other words, the alternative model (H_1_) predicts the observed data 10.8 times better than the null (Ho) model. According to the Bayes factor classification scheme (Figure 1), a BF_10_ of 10.8 is categorised as ‘strong’ evidence.

Figure 5 presents the results for the Bayes factor robustness test and shows that the Bayes factor remained stable across a range of Cauchy prior widths varying from small (*r* = 0.2) to ultrawide (*r* = 1.41) (Wagenmakers, Love, et al. 2018; Goss‐Sampson et al. 2020).

Table 2 shows that, using (Equation 1) (the odds form of Bayes' rule) a Bayes factor of 10.8 updated the prior model odds of 1:1 to the posterior model odds of 10.8:1 in favour of H_1_, and using Equation (2), the prior probabilities of 50% each for H_1_ and H_0_ were updated to 91.5% and 8.5%, respectively.

*Effect size and credible interval*: Figure 3 also presents the posterior probability distribution, summarised by a median standardised effect size, *d* = 0.406 (Cohen's *d* units) and a 95% (ETI) credible interval (or uncertainty) ranging from [0.132 to 0.684]. According to Cohen's *d* classification, the effect size, *d* = 0.406 corresponds to a small to medium effect for the educational intervention (Cohen 1992).

In absolute units, the median effect size is 4.1, and the uncertainty as expressed by the 95% credible interval ranges from [1.3 to 6.9].

Implications for nurse education: The Bayesian independent samples *t*‐test yielded a BF_10_ of 10.8, providing strong evidence in favour of the alternative hypothesis (H_1_), indicating that the educational intervention had a positive impact on examination scores. The updated model probabilities (91.5% in favour of H_1_) are deemed as sufficiently compelling evidence to support the conclusion that the educational intervention was effective. The median point estimate for the effect size (Cohen's *d* 0.406) suggests a small to medium improvement in student nurses' examination scores due to the intervention. Additionally, these findings suggest that implementing the educational programme could increase student nurses' examination scores by an estimated median absolute score effect size of 4.1 points with a credible interval [1.3 to 6.9].

## 12 Discussion

The Bayesian independent samples *t*‐test provides several statistically principled advantages that are not available to researchers relying solely on frequentist methods. It can be easily implemented using user‐friendly software packages such as commercially available IBM‐SPSS, freely available JASP or Jamovi software; the latter two have similar interfaces.

A frequentist independent *t*‐test was also conducted to illustrate its binary decision‐making approach with the Bayesian evidential method, the latter which can provide strength of evidence for H_1_ or H_0_ or inform about insufficient evidence. Table 3 presents the result of the frequentist (classical) independent *t*‐test, (two tail) which yielded a *p* value of 0.003 suggesting strong support to reject the null hypothesis. In contrast, the Bayesian independent *t*‐test result retained an 8.5% probability of support for the null hypothesis.

**TABLE 3:** Frequentist independent samples *t*‐test (two tail) for Examination score.

*t*	df	*p*	Mean diff	SE diff	Cohen's *d*	SE Cohen's *d*	95% CI for Cohen's *d*	
							Lower	Upper
3.037	198	0.003	4.350	1.432	0.429	0.145	0.149	0.709

*Note:* *p* = 0.003 is statistically significant at the 1% level.

Researchers may sometimes find that conclusions from the NHST approach and the Bayesian approach agree in simple situations, such as two‐group comparisons (Kruschke 2013). For instance, Table 3 shows a Cohen's *d* mean = (0.43) for the frequentist independent *t*‐test (two tail) and Figure 3 presents a median point estimate of (0.41) for the non‐directional Bayesian independent *t*‐test. When frequentist and Bayesian methods applied to the same data lead to similar conclusions, the Bayesian approach provides richer inferential information (Kruschke 2013). When the two approaches yield different conclusions, the Bayesian conclusion should take precedence (Dienes and Mclatchie 2018). With approximately normally distributed data, the mean and the median can be expected to be roughly the same due to the distributional symmetry.

In JASP software, the Bayesian Independent *t*‐test, parameterised with H_0_: *δ* = 0 indicates the use of a point null hypothesis. Researchers who wish to employ an interval null hypothesis can do so in JASP, by applying the ‘Equivalence Bayesian Independent Samples *T*‐Test’ which allows users to specify a small interval around zero, representing a non‐zero but small effect sizes around zero (Morey and Rouder 2011) deemed to be too small to be of practical or theoretical importance. According to (Aczel et al. 2020) using a point null instead of a very small interval null rarely lead to practically different conclusions. For example, an examination score increase of 0.1 Cohen's *d* (i.e., very small effect size) might not justify changing an educational programme given the costs of implementation.

This article applied the Bayesian independent samples *t*‐test in JASP software for both hypothesis testing (Bayes factor) and parameter estimation (effect size) which does not require programming skills due to its user‐friendly graphical interface. However, an alternative approach to Bayesian inference is available for users with programming skills. This method, popularised by Kruschke (2011, 2013) is implemented through freely available JAGS software and R programming language, which require users to write code to perform the analysis. This approach evaluates whether the null value falls within the most credible parameter values of the posterior distribution. It does so by comparing the highest density interval (HDI) to a region of practical equivalence (ROPE) around the null. The ROPE can be conceptualised as an interval null or small range of values around a specific value (typically zero) that are considered practically equivalent to zero. The HDI (Highest Density Interval) represents the range (typically 95%) of the most probable values within the posterior distribution for a parameter. If the 95% HDI falls entirely within the ROPE, the magnitude of the effect is considered negligible; if it falls entirely outside, the magnitude of the effect is meaningful; and if it overlaps the ROPE, the evidence is inconclusive.

## 13 Limitations

With Bayesian parameter estimation, the posterior is typically robust to prior variations provided that the sample size is sufficiently large and the prior is not overly narrow. On the other hand, model comparison can generate Bayes factors that are very sensitive to the choice of prior (Kruschke 2011). While the Bayes factor tends to remain relatively stable with reasonable prior variations (Rouder et al. 2009), the influence of the prior on the Bayes factor is retained even with large amounts of data (Stefan et al. 2022). Changing a prior is equivalent to changing the hypothesis (theory), so a change in the Bayes factor is expected (Jeffreys's platitude) (Etz et al. 2018). According to Schönbrodt et al. (2017), in the absence of strong prior information, a default (reference) prior is often recommended to minimise subjective or data‐driven prior selection. For the Bayesian independent *t*‐test, JASP provides a default Cauchy prior (standardised scale width 0.707) with the option to check a box to output the Bayes factor robustness across a range of plausible prior widths. This allows the researcher to assess whether variations in the prior width produce quantitative changes in the Bayes factor that impact the qualitative conclusion, that is, whether the Bayes factors are consistent when judged against the evidential categories. The numerous merits of using standardised effect sizes, include: aiding the establishment of the scale width of a prior, informing priors in new studies based on the results of previous studies, and combining results across studies in meta‐analysis. Using standardised units to establish priors is highly useful due to their generalisability across studies. Parameterising such priors in absolute units would be considerably more challenging. Currently, the Bayesian independent *t*‐test in JASP only reports the standardised effect sizes (e.g., Cohen's *d* units) without providing the corresponding absolute effect sizes or their credibility intervals. This can be a limitation when translating statistical evidence in practical situations. For example, knowing that an educational programme resulted in a median absolute increase of 4.1 examination points may be more meaningful to nurse educators than a standardised effect size, particularly when the absolute measurement scale is more familiar and interpretable. While the merits of standardised effect size are evident, supplementing it with absolute effect size results would further enhance the benefits of using JASP software. Additionally, in the current version of JASP at the time of writing (v0.18.3), the Bayesian independent *t*‐test output pane does not include the model probabilities, which is an intuitive metric for indicating support for each of the models.

## 14 Conclusions

Bayesian statistical techniques offer a powerful method for advancing nursing and midwifery science grounded on the axioms of probability. Bayesian methods allow for the formal updating of prior knowledge in light of new evidence, making them particularly well suited to support the development and testing of nursing and midwifery theories. Traditional dismissal of non‐significant *p* values in frequentist analysis has contributed to publication bias, as studies with non‐significant results are less likely to be published, thereby leading to the exclusion of these study results in meta‐analysis and inflating reported effect sizes (Cumming 2014). In contrast, Bayesian analysis supports publishers' decisions based on strength of evidence rather than binary significance thresholds. This enables researchers to publish null results with statistically principled justification, helping to reduce publication bias and improve the reliability of the evidence base (Dienes 2016). For nurses and midwives, the adoption of Bayesian methods enhances the ability to interpret findings in terms of the probability of an effect or theory being true, the strength of evidence and the precision of effect size estimates. This makes research findings more directly applicable to clinical practice and policy development. Given these advantages, we recommend the inclusion of Bayesian statistics in nursing and midwifery curricula to equip future professionals to engage in more transparent, robust and impactful research.

## Author Contributions

Helen Evelyn Malone: conceptualisation, visualisation, writing – original draft; writing – review and editing. Imelda Coyne: writing – review and editing.

## Disclosure

*Permission to Reproduce Material From Other Sources*: Permission was granted by Quintana and Williams (2018) for the use of a print screen representation of the Bayes factor classification scheme. Permission was granted by the JASP team (2024) for the use of print screens sourced from JASP Software.

## Ethics Statement

The authors have nothing to report.

## Consent

The authors have nothing to report.

## Conflicts of Interest

The authors declare no conflicts of interest.

## Data Availability

Data derived from public domain resources and available at:osf.io/4t9gn.
